# Gestational breast cancer in a patient with Crohn’s disease: two case reports

**DOI:** 10.1186/s13256-021-03224-3

**Published:** 2021-12-27

**Authors:** Mohammed Al-Arsan Al-Yaseen, Salah Aldin Haydar, Mousa Alali, Maher Saifo

**Affiliations:** 1 Faculty of Medicine, Damascus University, Fayez Mansour Street, P. O. Box: 222, Damascus, Syria; 2 Department of Oncology, Al-Bairouni University Hospital, Damascus University, Harasta M5, Damascus, Syria; 3 Faculty of Pharmacy, Alsham Private University, Damascus, Syria

**Keywords:** Crohn’s disease, Familial breast cancer, Lobular carcinoma, Chemotherapy, Gestation

## Abstract

**Background:**

Diagnosis of breast cancer during gestation is a rare occurrence. In addition, the diagnosis of breast cancer in a patient with Crohn’s disease is not common. We present a rare case of gestational breast cancer in a patient with Crohn’s disease, with a concurrent breast cancer diagnosis in her sister.

**Case presentation:**

A 31-year-old Syrian woman with Crohn’s disease was diagnosed with breast cancer at 30 weeks gestation; she received neoadjuvant chemotherapy during gestation. Incidentally, her 37-year-old sister was also diagnosed concomitantly with breast cancer. Both sisters underwent and successfully completed surgery and adjuvant therapy. At a 5-year review, both patients showed no signs of recurrence. The Crohn’s disease symptoms have also improved after chemotherapy, and the baby born after gestational chemotherapy is currently 5 years old with normal psychomotor development and without any congenital malformations.

**Conclusions:**

This case report highlights the impact of gestation on breast cancer outcomes, the possibility of giving chemotherapy during gestation, and the effect of chemotherapy on the symptoms of Crohn’s disease.

**Supplementary Information:**

The online version contains supplementary material available at https://doi.org/10.1186/s13256-021-03224-3.

## Background

Breast cancer is the most frequently diagnosed malignancy in females, accounting for around 30% of female cancers [1]. It is the leading cause of cancer death in women globally [1]. There are many risk factors for breast cancer such as female gender, increasing age, age at menarche, age at menopause, hormone replacement therapy, and reproductive factors such as nulliparity and late age at first full-term pregnancy among others. However, the strongest risk factor is family history; individual risk increases with the number of relatives affected with breast cancer and the decreasing age at which it was diagnosed [2].

The diagnosis and the treatment of breast cancer during gestation present a challenging situation for the patient, their family, and the physician. Because of the low incidence, clinical management decisions are limited to retrospective case series and case reports [3, 4]. Furthermore, the diagnosis of breast cancer in a patient with Crohn’s disease (CD) poses an added difficulty for managing its effect on breast cancer, as well as the effect of breast cancer on the treatment of CD.

This is a case report of two sisters who were concomitantly diagnosed with breast cancer; the younger sister with Crohn’s disease had breast cancer during gestation, and the elder sister was single with no reproductive history. Their parents are not relatives. This case report aims to detail our experience in diagnosing breast cancer during gestation and choosing the best treatment for both the health of the mother and the safety of the fetus.

## Case presentation

### Patient 1

**Fig. 1:**
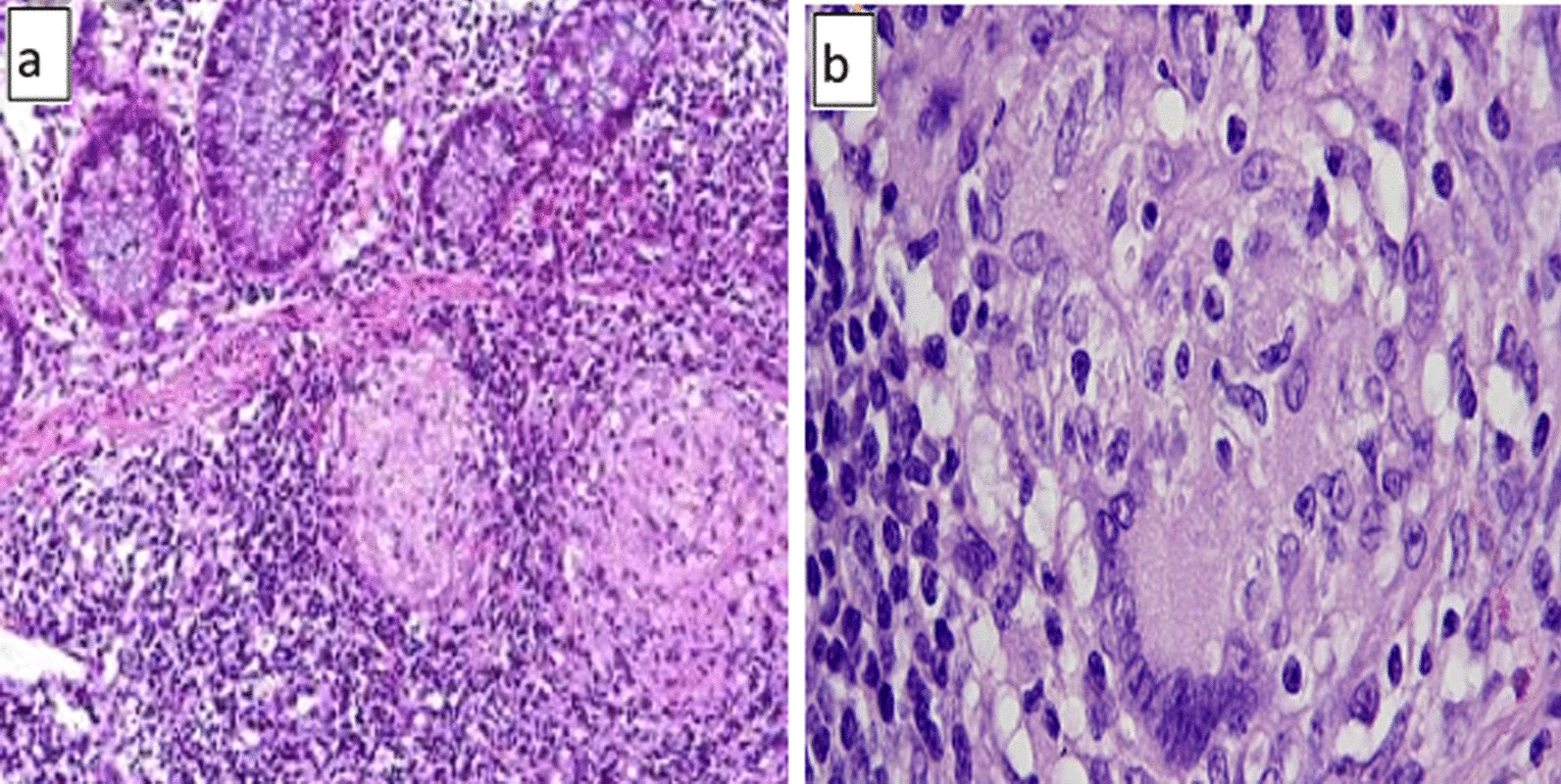
Histopathologic images of Crohn’s disease. **a**, **b** Medium- and high-power views showing inflammatory cell infiltration and granulomas with giant cells (hematoxylin and eosin)

A 31-year-old gravida 1, para 0 pregnant Syrian woman was admitted to Al-Bairouni University Hospital in May 2014 complaining of a palpable lump in her left breast. She is a housewife and at presentation was at 30 weeks gestation. She attained menarche at 13 years of age, and her menstrual periods are regular. She does not use tobacco or alcohol. She has no family history of breast cancer or other cancers. Her past medical history is significant for abdominal pain, bloody diarrhea, and mucus in her stools, which on colonoscopy and biopsies revealed a diagnosis of Crohn’s disease 1 year ago (July 2013) (Fig. 1). She was started with oral prednisone 20 mg daily, which was subsequently discontinued by the patient after 3 months because of inappropriate weight gain. About a month later, she got pregnant. She had taken some over-the-counter medications, including acetaminophen.

On physical examination, a firm, nontender mass in the upper outer quadrant of the left breast was found with no palpable axillary lymph nodes. Ultrasound of the left breast was suspicious for a malignant mass in the upper outer quadrant. Vital signs were within normal limits, the abdomen was soft, nondistended, and nontender without hepatosplenomegaly or masses. The neurological examination revealed intact cranial nerves and normal muscle strength in all extremities. Other clinical examinations were within normal limits. Laboratory results are as follows: Leukocytes: 6.60 × 10^3^/µL; neutrophils: 56%; hemoglobin: 11.5 g/dL; platelets: 301 × 10^3^/µL; urea: 31 mg/dL; creatinine: 0.74 mg/dL; alanine aminotransferase (ALT): 18 IU/L; aspartate transaminase (AST): 22 IU/L. The electrocardiographic findings and urinalysis were normal.

**Fig. 2:**
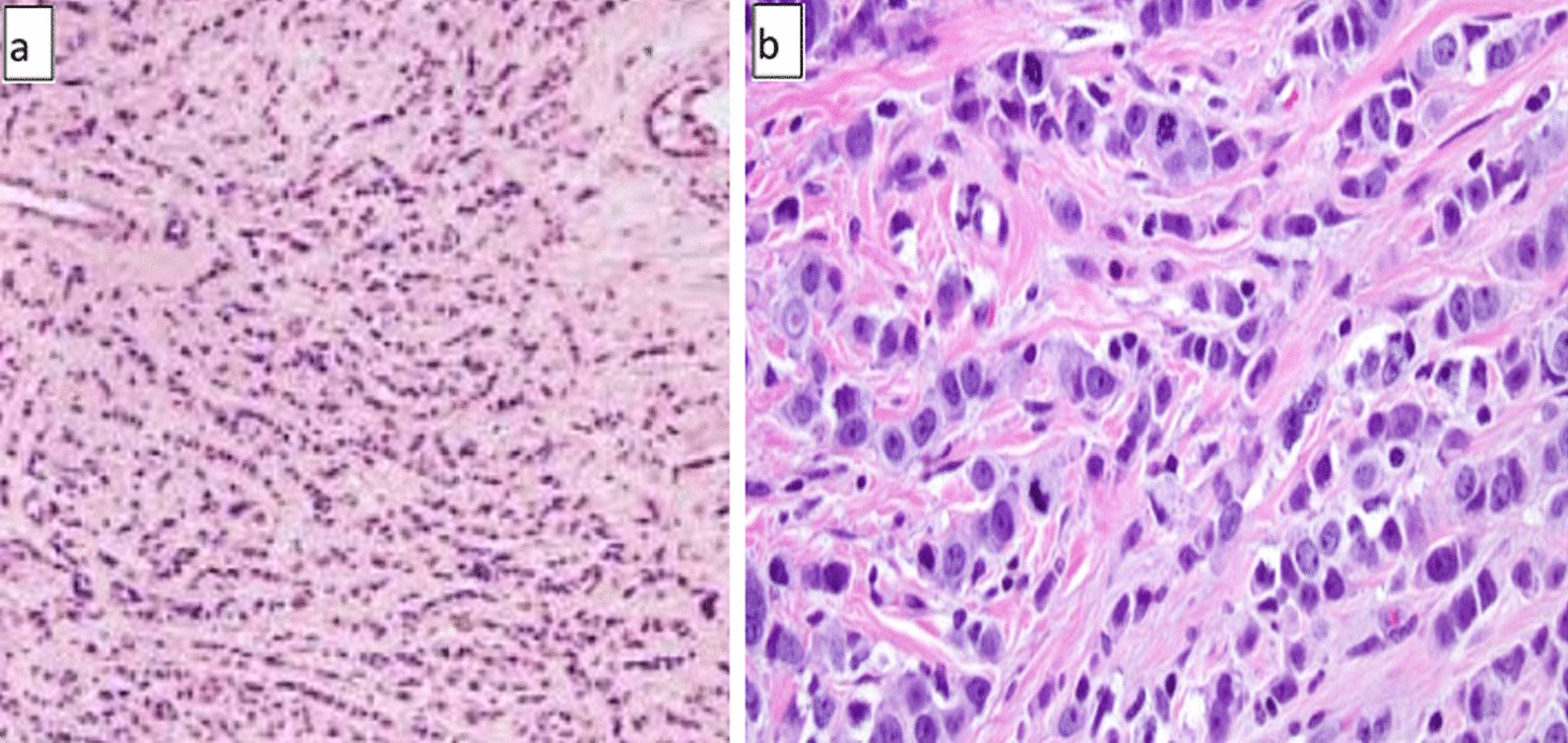
Histopathologic images of invasive lobular carcinoma. **a**, **b** Medium- and high-power views showing tumor cells organized in lines (hematoxylin and eosin)

The patient underwent an excisional biopsy that revealed a 6 cm mass (T3). The histopathology exam revealed an invasive lobular carcinoma (ILC) grade III (Fig. 2). Using immunohistochemistry (IHC), 30% of tumor cells stained positive for estrogen receptors (ER), negative for progesterone receptors (PR), and negative for human epidermal growth factor receptor-2 (HER-2) (+ 1). Ultrasound of the right breast was unremarkable. Furthermore, chest X-ray (with shielding) and abdominal ultrasonography showed no signs of metastatic disease (M0).

She received neoadjuvant intravenous chemotherapy, with two cycles of doxorubicin 100 mg and cyclophosphamide 1000 mg (AC); the period between the two cycles was 3 weeks. Then, she took a rest from chemotherapy for a month and delivered a live female infant appropriate for gestational age, with no congenital malformation, by a cesarean section. Postpartum, she underwent a left modified radical mastectomy and axillary lymph nodes dissection (MRM and ALND). The histopathology report showed that the margins were uninvolved and all the lymph nodes were free of malignant invasion (18/0) *N* = 0, which suggested a T3ypN0M0 score. After surgery, she continued the treatment with adjuvant chemotherapy containing the remaining third cycle of (AC) and four cycles of docetaxel 120 mg with 3 weeks interval during every dose of chemotherapy and underwent adjuvant radiotherapy to her left chest wall. Since then, she has remained on oral tamoxifen.

After more than 5 years of follow-up (February 2020), the patient is still alive and doing well without any signs of breast cancer recurrence. She has suffered from mild diarrhea for several days at a rate of 2–3 times per year and improved on occasional treatment without the need for cortisone or immune modulator drugs. Her child is now 5 years old with normal psychomotor development.

### Patient 2

A 37-year-old nulligravida Syrian woman was admitted to Al-Bairouni University Hospital in May 2014 complaining of a palpable lump in her right breast and nipple discharge. The patient took no medications and did not use alcohol or illicit drugs. Her past family medical history included a younger sister diagnosed with breast cancer and CD (patient 1). Her past medical history was otherwise unremarkable. She is not married and does not work, she attained menarche at 14 years of age and her menstrual periods are regular.

Upon physical examination, a firm mass in the upper outer quadrant of the right breast with a nipple discharge was found without any evidence of axillary lymphadenopathy. Vital signs were within normal limits; Abdominal, neurological, and other examination were unremarkable. Laboratory results were as follows: leukocytes: 7.86 × 10^3^/µL; neutrophils: 67%; hemoglobin: 12.6 g/dL; platelets: 268 × 10^3^/µL; urea: 24 mg/dL; creatinine: 0.61 mg/dL; ALT: 21 IU/L; AST: 25 IU/L. Electrocardiographic findings and urinalysis were normal.

Mammography and ultrasound of the right breast showed a suspicious mass in the upper outer quadrant. Therefore, she underwent an excisional biopsy for right breast mass, which revealed a 1 cm mass (T1). The histopathology exam revealed a poorly differentiated invasive ductal carcinoma (IDC). Using IHC, 20% of tumor cells stained positive for (ER), negative for (PR), and negative for (HER-2). The thoracic CT and bone scintigraphy did not show any signs of metastatic disease.

The patient underwent MRM and ALND. Negative margins were obtained with the surgery and only three lymph nodes were obtained, with an absence of tumor invasion (0/3) (*N* = 0). The final pathological result was T1N0M0 and a stage I tumor. After surgery, adjuvant intravenous chemotherapy was administrated with three cycles of fluorouracil 850 mg, doxorubicin 85 mg, and cyclophosphamide 850 mg (FAC); and three cycles of docetaxel 130 mg with a 3-week interval. After that, she was treated with adjuvant radiotherapy. Since then, she has remained on oral tamoxifen. After more than 5 years of follow-up (February 2020), she is still alive and doing well without any signs of recurrence (Additional file 1).

## Discussion and conclusions

This is the first case report of concomitant breast cancer diagnosis in sisters, in which one was pregnant and incidentally had a prior diagnosis of Crohn’s disease. Reviewing the literature (PubMed and Google Scholar search, June 2021), we found no identical cases. However, few similar cases reported breast cancer in two sisters, such as Wang *et al.* [5]

Diagnosis of breast cancer during gestation is rare and occurs in approximately 15–35 per 100,000 deliveries, and has increased recently because of childbearing delay [3, 4]. The risk of breast cancer in women who have their first child after the age of 30 years (patient 1) is about twice that of women who have their first child before the age of 20 years Hormonal changes during gestation (e.g., engorgement and hypertrophy) make the physical examination more difficult and reduce the sensitivity of breast imaging. As a result, these changes delay the identification of suspicious masses. Ultrasonography is a useful tool for initial evaluation. Biopsy should be used as a diagnostic tool and cannot be delayed until after delivery [6, 7].

Gestational breast cancer includes medical, psychological, and ethical issues, and is still a problem for the oncologist, surgeon, and gynecologist. Chemotherapy with AC or FAC is safe after the 14th week of gestation. Because our patient was in the 30th week of gestation, she was given neoadjuvant AC. Radiotherapy and hormonal therapy were postponed until after birth [6–8]. The same approach was followed by Schad *et al*. and Nye *et al*., suggesting the safety and efficiency of this therapeutic plan [9, 10].

CD is a risk factor for intestinal cancers, but there are inconclusive results whether or not it is a risk factor for breast cancer or other malignancies. However, some studies have reported that females with first-relative CD patients have a higher risk of developing breast cancer [11, 12]. No common genetic background has been established between CD and breast cancer [13]. Many studies suggest that cancer treatment with cytotoxic chemotherapy may induce and maintain CD remission by causing cell death or preventing cell division in rapidly dividing cells such as T lymphocytes or malignant cells, yielding anticancer and immunosuppressive effects [14]. This may justify the absence of any flare in the patient after 5 years of follow-up.

The family history of breast cancer is an important risk factor: the risk of the disease will increase with the number of relatives affected and their ages when they were diagnosed; the younger the age at diagnosis of breast cancer, the greater the risk. The risk of breast cancer is two or more times greater if the patient has a first-degree relative (mother, sister, or daughter) who was diagnosed with breast cancer before the age of 50 years [2, 15]. In our case, breast cancer was diagnosed in two sisters, both of whom were less than 40 years old, which strongly indicates the presence of familial breast cancer.

Familial predisposition derives from the interaction of genes and environment/lifestyle choices. Several genetic mutations have been identified as high-risk factors for breast cancer. Mutations in the tumor suppressor genes *BRCA1* and *BRCA2* are responsible for the majority of hereditary breast cancer cases. Women who inherit a *BRCA1* or *BRCA2* mutation face a substantial risk of developing breast cancer, estimated at 72% and 69%, respectively [16]. Mutation in *BRCA1* and *BRCA2* are also responsible for the increased risk for developing early onset breast cancer and familial ovarian cancer, the early onset type of breast cancer tends to have high intensity and be bilateral. *BRCA1* and *BRCA2* are usually seen in patients with a family history of breast cancer. In addition, patients who carry *BRCA1* and *BRCA2* mutation have a higher risk for developing other types of cancers such as colon, prostate, pancreatic, melanoma, and gastric cancers. The identification of genes associated with a predisposition to breast cancer, such as *BRCA1* and *BRCA2* is an important tool to increase surveillance and effective prophylactic intervention [17].

Unfortunately, *BRCA* and other genetic tests have not been conducted here because of financial reasons. Thus, we strongly advised the two patients to do *BRCA1* and *BRCA2* tests as soon as they can, especially because the two patients are first-degree relatives.

Determination of biomarker status, including ER, PR, and HER2 status, is essential for newly diagnosed breast cancer. They also have a crucial role in choosing the most appropriate treatment option. ER is used in identifying patients with early onset breast cancer to determine if they need tamoxifen or an aromatase inhibitor in their treatment regimen (patient 1). Administration of tamoxifen as an adjuvant therapy for 5 years in patients with +ER was found to decrease recurrence by approximately 50% [18].

In conclusion, this reports a rare case of familial breast cancer in two sisters at the same time; the younger one had CD and was diagnosed during gestation. This highlights the association of gestation with a more advanced clinical stage of breast cancer, the possibility of giving chemotherapy during gestation, and the positive impact of chemotherapy on the symptoms of Crohn’s disease.

## Supplementary Information

**Additional file 1: Figure S1.** The timeline for patient 1. **Figure S2.** The timeline for patient 2.

## Data Availability

The data that support the findings of this study are available from the corresponding author upon reasonable request.

## Abbreviations

CD: Crohn’s disease
ILC: Invasive lobular carcinoma
IHC: Immunohistochemistry
ER: Estrogen receptor
PR: Progesterone receptors
HER-2: Human epidermal growth factor receptor-2
AC: Doxorubicin and cyclophosphamide
MRM and ALND: Modified radical mastectomy and axillary lymph nodes dissection
IDC: Invasive ductal carcinoma
FAC: 5-Fluorouracil, doxorubicin, and cyclophosphamide
