# Supplementary material for: Gestational breast cancer in a patient with Crohn’s disease: two case reports
Source: J Med Case Rep. 2021 Dec 27;15:633. doi: 10.1186/s13256-021-03224-3 (PMC8711207; doi:10.1186/s13256-021-03224-3)
Supplement: Supplementary file 1 — Additional file 1: Figure S1. The timeline for patient 1. Figure S2. The timeline for patient 2. [file 13256_2021_3224_MOESM1_ESM.pdf]

The timeline of " Gestational breast cancer in a patient with crohn's disease: a case report of two sisters "

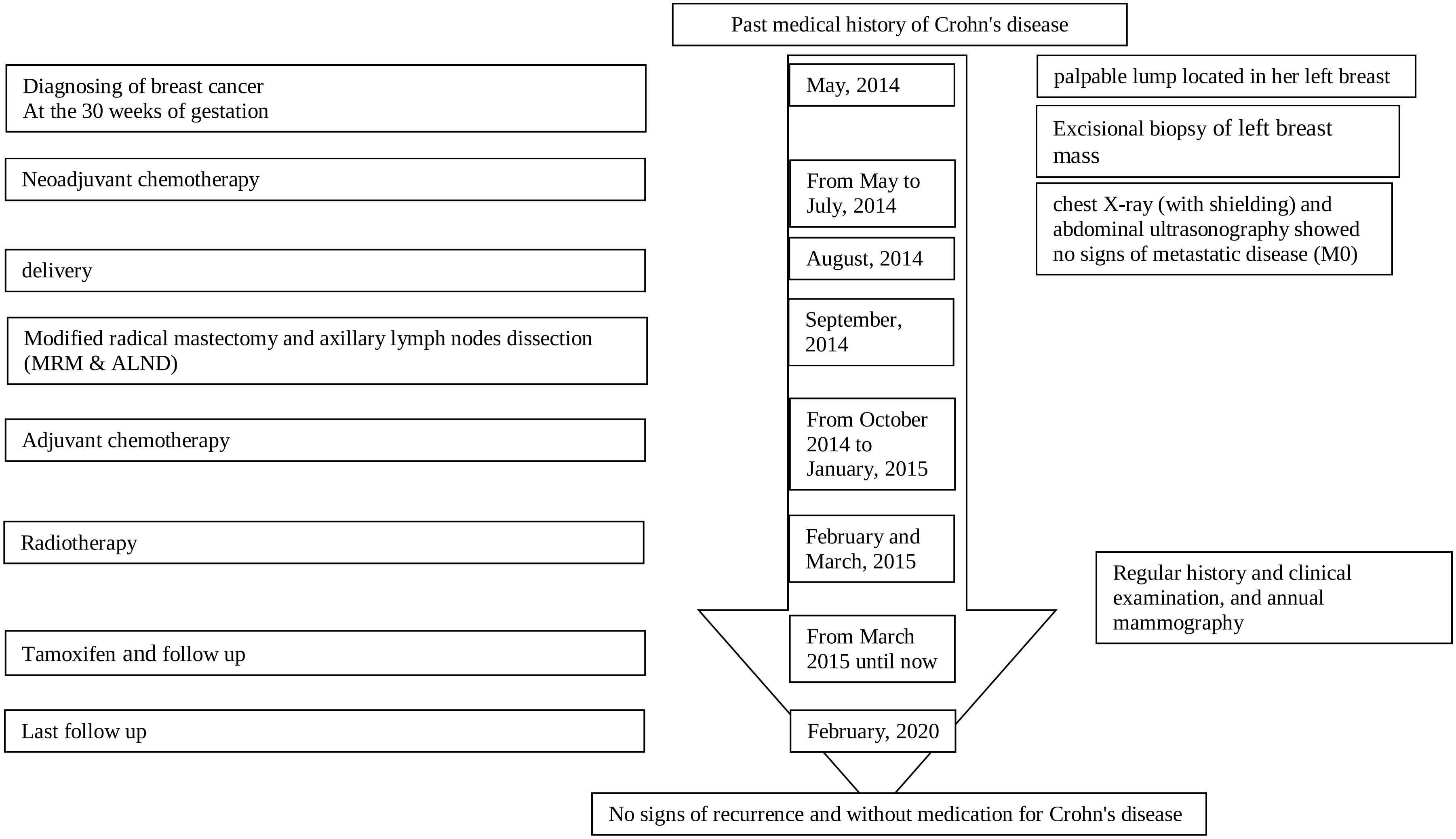

Figure 1. The timeline for patient 1.

The timeline of " Gestational breast cancer in a patient with crohn's disease: a case report of two sisters "

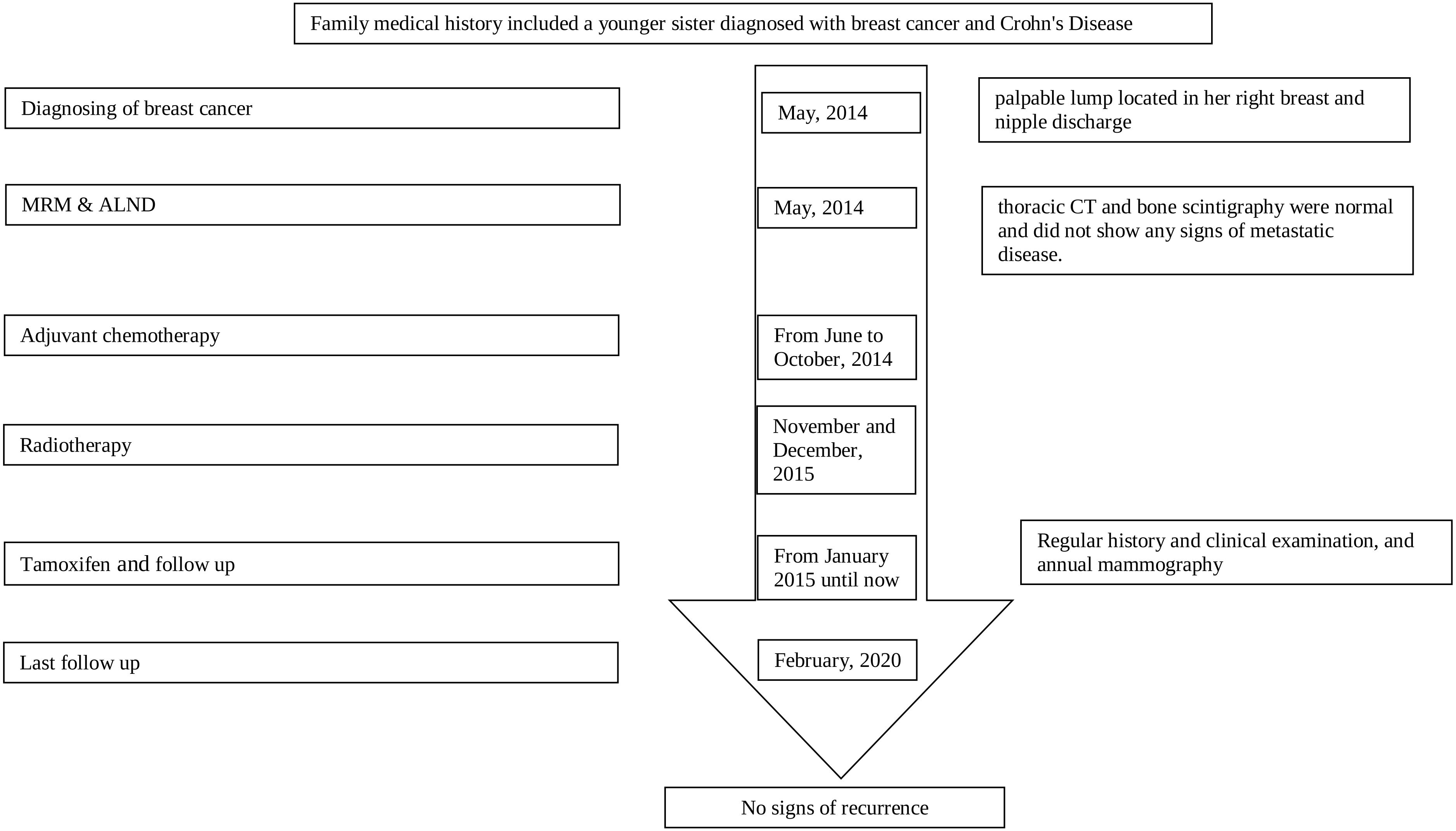

Figure 2. The timeline for patient 2.
